# Expert Consultation: Factors Influencing End-of-Life Decision-Making for Dairy Cattle Across the United States Supply Chain

**DOI:** 10.3390/ani14223311

**Published:** 2024-11-18

**Authors:** Lily Edwards-Callaway, Brianna McBride, Erica Machuca, Lauren Dean, Kira Sayre, Catie Cramer, Noa Román-Muñiz, Kayleigh Keller, Lorann Stallones, Diego Manriquez

**Affiliations:** 1 Department of Animal Sciences, Colorado State University, Fort Collins, CO 80523, USA; brianna.mcbride@colostate.edu (B.M.); erica.machuca@colostate.edu (E.M.); lauren.dean@colostate.edu (L.D.); kira.sayre@colostate.edu (K.S.); catie.cramer@colostate.edu (C.C.); noa.roman-muniz@colostate.edu (N.R.-M.); diego.manriquez_alvarez@colostate.edu (D.M.); 2 Department of Statistics, Colorado State University, Fort Collins, CO 80523, USA; kayleigh.keller@colostate.edu; 3 Department of Psychology, One Health Institute, Colorado State University, Fort Collins, CO 80523, USA; lorann.stallones@colostate.edu

**Keywords:** animal welfare, dairy supply chain, end-of-life, euthanasia, fitness to transport

## Abstract

The end-of-life (i.e., on-farm euthanasia and culling, including fitness for transport to slaughter) of a dairy cow or calf is preceded by a series of decisions often influenced by many complex factors. The aim of this study was to utilize the opinions of experts with roles in the dairy supply chain to identify factors that influence end-of-life decision-making for dairy cattle. Twenty experts serving in supporting roles in the dairy supply chain participated in a survey containing 17 open-ended questions. Of the participants, 55% (*n* = 11) identified as veterinarians and the remaining 45% (*n* = 9) participated in other segments of the dairy supply chain. Twelve themes were identified in the qualitative analysis of survey responses. The themes included: Training and Resources, Cattle Health Management, Decision-Making Criteria, Company Culture, Personal Beliefs, Human Well-Being, Animal Welfare, Economics, Guidelines and Inspections, Consumer Perceptions, Supply Chain Considerations, and Communication. Experts indicated that training programs should address euthanasia or fitness-for-transport criteria, the knowledge of common health conditions, and information about what happens to cattle after leaving the dairy. Additional influencing factors included personal beliefs, economics, company culture, and supply chain considerations.

## Simple Summary

Making end-of-life decisions for dairy cattle (i.e., euthanasia and fitness for transport) is a complex topic with a significant impact on dairy cattle welfare and the sustainability of the entire dairy supply chain. In this study, experts serving supportive roles in the dairy industry identified factors that influence end-of-life decision-making for dairy cattle. The results highlight twelve themes, providing insight into how end-of-life decisions can be improved. The entire supply chain (i.e., dairy farms, transporters, auction markets, and processors) has a responsibility to work collaboratively to ensure end-of-life decisions are being made with dairy cattle welfare in mind.

## 1. Introduction

Ensuring animal welfare throughout the livestock production chain is a responsibility of all stakeholders and an ever-growing expectation of consumers [1,2]. End-of-life decision-making (i.e., proactive culling, timely euthanasia, fitness for transport, etc.) for dairy cattle has been identified as a high-risk challenge area in need of attention [3,4,5,6]. Dairy cows provide different commodities, i.e., milk and meat, at two points in their life. In 2023, over 3 million dairy cows were slaughtered at federally inspected plants [7]. A portion of culled dairy cattle are shipped directly to slaughter annually, but this represents a smaller portion of the population as compared to cattle that are sent to a livestock sale or auction directly from the dairy (i.e., dairy farm); the National Animal Health Monitoring System (NAHMS) reported that 92% of operations sampled marketed some of their culled cows through a livestock auction or market and 37% sent some cows directly to slaughter [8]. Making proactive culling decisions can prevent high-risk animals from entering the market by selling animals that are still able to withstand the stressors that may occur after leaving the farm prior to arriving at a slaughter plant [3,9]. This final decision at the dairy is critical for a dairy cow’s welfare as she transitions out of the milking herd and makes her journey into the beef supply chain.

Despite there being animal care guidelines established by each industry sector that provide guidance for making euthanasia, culling, and fitness-for-transport decisions (e.g., the American Association of Bovine Practitioners (AABP) [10,11]; Farmers Assuring Responsible Management (FARM) [12]; the Livestock Marketing Association (LMA) [13]; and The Meat Institute [14]), there is evidence suggesting that decisions made throughout the supply chain do not always prioritize dairy cattle welfare. Instead of animals that have no chance of recovery being culled early or euthanized, some of them are shipped to slaughter in an unfit condition. The results of the 2022 National Beef Quality Audit, a benchmarking activity sponsored by the National Cattlemen’s Beef Association (NCBA), indicated that 64% of dairy cows had a defect (e.g., a swollen joint or foot abnormality), 23% were “too thin”, and 8% had a moderately impaired mobility [15] upon arrival at the slaughter facility. Similarly, Vogel et al. [16] characterized the conditions of cull cows arriving at slaughter plants in the United States as part of a global study and reported that 9% of cows had one or more of the measured criteria which included the following: severe lameness, a low body condition score, a poor udder condition, severe uterine prolapse, cancer eye, malaise, wounds, active parturition, a nervous system disorder, and a non-ambulatory condition. More recent research in Canada demonstrated similar results; Stojkov et al. [17] reported that 30% of cows observed at sale barns in Canada were not fit for transport, suggesting that some poor decisions for shipment were made for the dairy cattle.

End-of-life decisions for dairy cattle are challenging to make and are influenced by many, and often competing, social–ecological factors (e.g., interconnected factors representing individual, interpersonal, organizational, communal, and societal influences). Although there has been some research exploring factors that influence on-farm euthanasia decision-making with dairy caretakers and veterinarians [18,19,20,21,22,23], there is limited comparable research across other supply chain stakeholders (e.g., auction market employees and transporters). Similarly, although fitness for transport is a key component of cattle industry best management practices (Beef Quality Assurance (BQA)) [12,24], there are even fewer studies exploring stakeholder knowledge and decision-making around fitness for transport, and most of those studies have been conducted outside the United States [25,26,27].

One of the challenges with improving end-of-life decisions for dairy cows and calves is that there are many stakeholders and the limitations, challenges, and incentives at each point in the supply chain differ. The supply chain is complex. The ownership of and responsibility for dairy cattle may change multiple times during this final end-of-life (i.e., marketing) stage as dairy cattle could transfer through dairies, calf ranches, auction markets, multiple transporters, and slaughter plants. This change in ownership is driven by financial transactions and there are often economic incentives for moving animals through the system regardless of their condition [4]. A comprehensive understanding of these diverse obstacles and needs, currently lacking in research, is crucial for crafting holistic solutions for improving dairy cattle welfare at this critical juncture that are both effective and practical. Past research has successfully employed expert consultations to initiate the investigation of complex problems related to dairy cow welfare [3,28]. This project’s objective was to utilize expert consultation to identify factors that influence end-of-life decision-making for dairy cattle in the United States’ dairy systems. Although the authors feel that these challenges could be relevant across areas of the world, there are 9 million milk cows in the United States [29] and the majority of milk cows are on farms with a milk cow herd size of over 1000 [30], providing a perspective on the expert opinions shared in this study.

## 2. Materials and Methods

Prior to project initiation, Colorado State University’s Institutional Review Board (IRB #5080) approved this study.

### 2.1. Survey Development and Content

Members of the research team created the survey and adapted questions from Velosa [31]. The first section of the survey comprised 17 free-response questions related to factors that may influence the euthanasia and fitness for transport of dairy calves and cows that may be located on dairies, on calf ranches, at auction markets, in transport vehicles, or at slaughter plants (Table 1). Participants were instructed to complete sections which they felt comfortable answering and had experience in and were not required to answer those in which they did not feel they had sufficient experience to address. The second section included four multiple choice questions related to demographics (e.g., gender, area of work, degree(s) earned, and years of experience).

### 2.2. Study Population and Distribution

In November 2023, thirty experts across the dairy supply chain in the United States were contacted to participate in this survey; at least one author was acquainted with each individual contacted. The individuals were selected by members of the research team based on their experience and area of expertise across different sectors of the dairy supply chain and associated groups; these individuals were identified as involved in or supervisors of end-of-life decision-making and served in roles supportive to producers, consignors, and processors. The groups represented included academia (including extension and academic veterinarians), practicing dairy veterinarians, representatives from industry organizations, and individuals working with slaughterhouses. The survey was distributed through email, as an attached document to fill in, to these individuals, containing a brief description of the project, consent information, and the survey to be filled out electronically and returned. Reminders were sent weekly for three weeks to those who had not turned in a survey. The surveys were de-identified (i.e., names, emails, and any identifying information was deleted) upon receiving the email attachment, and all emails were permanently deleted. No incentives were provided, and consent was received via email with the attached completed survey.

### 2.3. Qualitative Analysis Methodology

Demographics were summarized in Microsoft Excel (Microsoft Corp., Redmond, WA, USA) and free-response answers were collated in Microsoft Word (Microsoft Corp., Redmond, WA, USA). The researchers determined that they had reached data saturation when incoming surveys provided minimal additional insights beyond the information already gathered, indicating that the collected data were sufficient to address the study objectives. This occurred after three-quarters of the returned surveys had been received, but the authors chose to include the additional surveys received after this time as well.

This study employed an inductive framework approach to analyze the qualitative data [32]. This approach allowed for themes to be identified from the data, rather than imposing a predetermined theoretical framework. An interpretivist ontology guided the analysis, focusing on understanding the subjective meanings that the participants attributed to their experiences. A constructivist epistemology was adopted, acknowledging that knowledge is socially constructed and influenced by the researcher’s perspective. This approach aimed to provide a rich and nuanced understanding of the participants’ experiences and the social context in which they occur. A group of authors and other researchers that have participated in other related projects reviewed the survey responses and familiarized themselves with the data. They then assigned initial codes within the responses. The group determined a list of initial themes that identified patterns across the codes. A group of three researchers (CC, BM, and EM) then refined, defined, and named the themes and conducted a comprehensive coding process. Each survey response was systematically assigned to one or more relevant themes, ensuring that the data were thoroughly categorized and analyzed within the established framework.

The three researchers assigned themes individually and then met to discuss the results. When disagreements occurred, the researchers discussed the response and came to an agreement for the coding. One coder was an assistant professor (PhD), with a focus on livestock behavior and welfare, currently conducting research on dairy calf health and transport. The second coder was a research associate (BS) in Animal Sciences, focusing on dairy cattle welfare. The third coder was working on her master’s degree in Animal Science, with a focus on livestock behavior and welfare. The researchers who conducted the qualitative analysis acknowledged that their personal experiences and backgrounds may influence their understanding of the research topic. While aware of potential biases, the researchers strived to be mindful of these and to actively seek out diverse perspectives to ensure that the research was inclusive and representative.

## 3. Results

### 3.1. Demographics

The survey was sent to 30 individuals, and 20 surveys were returned for a 67% response rate. Over half of the respondents worked directly in the supply chain (*n* = 12, 60%) but all worked directly with one or more sectors of the dairy supply chain. Of those returned, 40% (*n* = 8) were involved in academia, with six of these individuals being veterinarians and some explicitly indicating they had roles in extension; 25% (*n* = 5) were practicing veterinarians in the dairy and calf ranch sectors, 20% (*n* = 4) were representatives of supportive industry associations, and 15% (*n* = 3) were directly involved with a processor/slaughter facility. More than half (60%, *n* = 12) of the experts had 10+ years of experience in their sector of expertise, 20% (*n* = 4) had 4–10 years of experience, 15% (*n* = 3) had 1–3 years of experience, and one respondent did not answer this question. Women comprised 70% (*n* = 14) of the expert responses and men made up 30% (*n* = 6). Fifty-five percent (*n* = 11) of the respondents held a Doctor of Veterinary Medicine (DVM) degree, 30% (*n* = 6) held a doctorate degree, 25% (*n* = 5) held a master’s degree, and 15% (*n* = 3) held a bachelor’s degree.

### 3.2. Qualitative Results

Respondents were asked 17 open-ended questions regarding topics, challenges, factors, and misconceptions that influence timely euthanasia and fitness-for-transport decisions for dairy cattle throughout the supply chain. Twelve themes were identified. All theme definitions with examples of survey responses within the theme are presented in Table 2. Each theme is discussed below.

#### 3.2.1. Training and Resources

Respondents expressed Training and Resources as a need, with specific examples of types of training and resources, but also as a challenge or a barrier, in that there is a lack of the appropriate type of training and resources available to employees. Some responses were about general training needs such as “*employees need training in identifying conditions that require euthanasia, authority from their employer to make those decisions and also the skills and tools to carry out the procedure*”. Other responses were more specific and included specific types and topics of training needed for employees to effectively make both euthanasia and fitness-for-transport decisions, including recognizing normal versus abnormal health conditions and understanding prognoses; these types of responses were often identified with the Cattle Health Management and Decision-Making Criteria themes. Different modes of training and material delivery mentioned included “*visual pictures of body condition scoring, BQA flow chart of timely euthanasia decisions*”, “*visual reinforcement and reminders*”, “*posters*”, “*videos*”, “*training via in-person*”, and “*workshops*”. This theme was often found with the theme of Communication. For example, respondents emphasized the importance of “*making sure trainings are in the caregivers primary language*” to ensure the understanding of the information.

The challenges that respondents identified related to Training and Resources were related to employees “*not having the right equipment*” for euthanasia, not having the “*knowledge of how to use [euthanasia] equipment*”, and an overall “*lack of training and access to euthanasia equipment*”. There was also a continued mention of explaining the “why” during training programs; for example, one respondent stated that “*the ‘we’ve always done it this way’ mentality can be a difficult hurdle to overcome. Changing minds (and hearts) for adult learners typically requires instilling an understanding of the ‘why’ rather than simply dictating the “what*”.

#### 3.2.2. Cattle Health Management

The theme of Cattle Health Management included mentions of animal health, disease, treatment, veterinary evaluation, and many topics, both specific and broad, related to cattle care from a health perspective. This theme was frequently identified in responses to questions about required knowledge, skills, and training that caretakers should have for making decisions about and performing actions related to euthanasia and fitness for transport. For example, respondents stated that employees should understand how to “*recogniz[e] normal versus abnormal health conditions*”, “*signs, treatment and monitoring of progress for the following diseases: diarrhea, pneumonia, sepsis, meningitis, musculoskeletal injuries*”, and “*metritis/RP, milk fever, pneumonia, ketosis, DA*” [RP = retained placenta; DA = displaced abomasum]. Respondents frequently discussed the need for employees to understand prognoses for diseases or conditions to determine appropriate timelines for making decisions (e.g., “*likelihood/timeline of recovery*”).

#### 3.2.3. Decision-Making Criteria

This theme included responses that referenced to specific criteria that employees could (or should) use in the end-of-life decision-making process. Many of the responses indicated the importance of using scoring systems as decision-making criteria, such as “*body condition scoring*”, “*lameness*”, and “*mobility scoring*”. Respondents also mentioned “*animal physical assessment*” and “*animal history*” as important components of the decision-making process. Further, understanding and following drug withdrawal times was mentioned as a critical topic relative to understanding fitness-for-transport decisions through comments such as “*confirming girls are not waiting on a beef withdrawal*” and “*withdrawal times observed for meat and milk*”. These responses demonstrate the importance of employees understanding not only on-farm implications but also how these decisions can impact the entire supply chain.

The Decision-Making Criteria theme also included more general statements about the need for employees to be trained in decision-making itself. For example, the respondents recognized that training programs should specifically detail “*how to decide to perform euthanasia*”, “*how to make the right decision for each [animal or condition]*”, and how “*employees should be able to identify the best course of action for the animals[‘] welfare*”. Many pointed out the need for management to outline caretakers’ roles related to the decision-making process: “*identify[ing] who is responsible for making the decision, how often they will make it, and if that same person is also responsible for executing the decision*”. Additionally, respondents said that all personnel should know the “*chain of command in decisions*”, how to use “*decision making tree[s]*”, and “*contact information for appropriate personnel in case of emergencies*”.

Additionally, the Decision-Making Criteria and Cattle Health Management themes were often identified together. For example, one respondent said “*employees on dairy farms should be able to recognize common animal health conditions to make informed decisions as to when to transport an animal or see treatment for them first*”, identifying the importance of using cattle health outcomes as indicators to make informed decisions.

#### 3.2.4. Company Culture

This theme included any comments related to an organization’s structure, values, beliefs, and role in setting expectations for employees in the areas of both euthanasia and fitness for transport. Respondents identified that Company Culture can heavily influence end-of-life decision-making, both positively and negatively, and thus it was identified as both a barrier and a motivator. The responses were more related to the negative impacts that Company Culture could have rather than exploring it as a positive contributor to the work environment. Respondents discussed employees’ hesitancy to euthanize animals out of “*fear [of] losing [their] job*” and delayed decision-making due to not knowing “*who makes the decision and who is responsible for the cost*”. The respondents continually highlighted the importance of clear expectations from the company or management on the roles and responsibilities of employees related to the topics of decision-making around euthanasia and fitness for transport. They also identified that sometimes there is pressure from management to make certain decisions that may not always be congruent with what employees feel is in the best interest of the cattle. Responses with this theme were often also coded as Communication. Respondents noted that communication breakdown within a company structure could lead to some unclear expectations. Additionally, it was identified that experts felt that the lack of clarity and challenges with communication around this type of decision-making could lead to “*fear of reflecting on job performance*” and “*fear of being blamed for the animal’s condition*”, leading to a challenging work environment.

#### 3.2.5. Personal Beliefs

This theme included statements related to employees’ culture, religion, beliefs, opinions about their role in making decisions, and emotions towards animals. Personal beliefs were identified as factors that influence how, when, and if caretakers make decisions about euthanasia and fitness for transport. Responses such as “*we as humans and as cultures all process death differently*” highlighted how personal beliefs influence decision-making. Responses in the Personal Belief theme were often coded in tandem with the Animal Welfare theme, detailing how an employee’s personal beliefs towards animals such as “*empathy*” or “*lack of empathy*” could impact decision-making. The concepts of caretakers viewing “*euthanasia is a failure*” or “*… as criticism to their work*” were also suggested as factors that could delay euthanasia. Some respondents highlighted the difficulty for individuals who are tasked with caring for animals as part of their job responsibility to also have to make decisions about euthanizing them, even if that is the best option for the animals’ welfare; for example, some respondents shared potential beliefs that employees may have: “*[my] job is to save them not kill them*”, “*if there is still a chance, she is going to recover we have to keep trying*”, and “*I need to give them a chance to get better*”.

#### 3.2.6. Human Well-Being

Responses characterized as Human Well-Being were primarily related to human safety. Although an important consideration, this theme was not found extensively in the survey responses. It was mostly related to answers regarding resources and training, with individuals mentioning the need for knowing “*…how to be safe, firearm safety*” as it relate to performing euthanasia. Additionally, there were some comments extending to the safety of consumers as it relates to food safety; some respondents indicated that employees needed to understand aspects of “*Food Safety—how to ID animals who must be must be removed from the food chain in a timely manner*” as it specifically relates to safety impacts on the supply chain.

#### 3.2.7. Animal Welfare

This theme was focused on the mention of anything that was related to an animal’s quality of life and its well-being. Responses within this theme were found in answers to questions about factors that impact decision-making. Many comments were categorized as both Animal Welfare and Personal Beliefs as it is an individual’s feelings about animals that may impact their decisions about euthanasia and fitness for transport. The experts noted that sometimes misconceptions about how an animal is feeling (or not feeling) may exist and impact decisions, such as “*she is not suffering*”, “*animals don’t suffer like people do*”, “*animals don’t feel pain and stress like humans*”, animals “*have higher pain tolerance*”, and animals “*suffer less*”. One respondent recognized that a person’s feelings towards an animal may make euthanasia a more difficult decision; for example, “[caretakers] *have affection understandably for their cows and they just plain don’t like to euthanize or want to see them euthanized*”.

#### 3.2.8. Economics

Economics was mentioned as both an incentive (i.e., a motivator) and a disincentive (i.e., a barrier) that impacted the implementation of practices for timely euthanasia and fitness for transport. Respondents shared how financial incentives could work to influence the decisions of employees and operations by rewarding timely euthanasia and fitness-for-transport decisions while penalizing poor practices. For example, experts shared the following incentives (or disincentives) that could influence decisions: “*bonuses tied to having no down animals*” and receiving a “*penalty if an animal shouldn’t have been shipped or should have been euthanized*”. However, experts specifically emphasized that employees should not be penalized for euthanizing an animal when warranted; for example, one suggestion was that “*employees are rewarded for the quality of animals they raise, not the quantity*” and that there should be “*no deductions for euthanasia or non transported animals to the employees*”. Respondents shared how the fear of “*direct economic loss*” if an animal dies on site or during transport can impact practices, i.e., if cattle shipped to slaughter in a poor condition for the fear of the financial loss. Furthermore, economic constraints such as a lack of staffing, facilities, or resources can hinder the ability to identify and manage issues related to timely euthanasia and fitness for transport. One expert shared that some individuals may believe that “*money and getting paid supersede everything else*”, “*if the goal is to make money then we don’t euthanize on site unless we absolutely have to*”, “*selling an animal at any price is more profitable that euthanizing it*”, and “*compromised animals are still worth money*”. Although not mentioned frequently, the challenges and costs associated with carcass disposal when euthanizing on-farm was mentioned. These comments capture how economic motivators can make an end-of-life decision complicated to make depending on the individual’s and company’s prioritization of values.

#### 3.2.9. Guidelines and Inspections

This theme included comments about common industry guidelines, regulations, and audits and was found in response to questions about what skills and knowledge employees should have. The lack of regulations and consistent guidelines related to these topics was also identified as a challenge. Associated with the Training and Resources theme, many respondents mentioned many industry programs that focus on both dairy and beef cattle care such as “*BQA*, *BQAT*, *FARM*”, “*AABP*”, and “*CCQA*” (BQA, Beef Quality Assurance; BQAT, Beef Quality Assurance Transportation, BQAT; FARM, Farmers Assuring Responsible Management; AABP, the American Association of Bovine Practitioners Association; CCQA, Calf Care Quality Assurance). These programs were mentioned as beneficial resources to use for training caretakers and for protocol development.

Respondents shared that a lack of regulations and guidelines, and not enforcing existing regulations, could impact the implementation of timely euthanasia and fitness-for-transport practices. This theme was often found with Company Culture and Economics. One respondent stated, “*it is up to the moral compass of the owner and leadership from the top down… to really regulate and implement practices on farm*”. Respondents shared that there is a need for greater restrictions and penalties such as “*fines for not following the rules*”, “*loss of market due to repeat violations*”, and “*USDA write-up and potential plant shut down*” as mechanisms for regulations to create positive change in the condition of dairy cattle that are shipped to and/or passed for slaughter. One expert pointed to regulations in another country as an example, by sharing that “*unfit animals in Canada are not allowed to be hauled anywhere and they can be in the U.S.*”, identifying that stricter stances of expectations have been set in countries outside the United States.

#### 3.2.10. Consumer Perceptions

This theme encompassed comments related to consumers’ perceptions, thoughts and ideas about the dairy supply chain. Many of the responses related the risk of poor decision-making around euthanasia and fitness for transport to bad publicity and negative consumer reactions (i.e., “*bad publicity and boycotts or bans*” and “*euthanasia decisions might affect the social perception of the dairy industry affecting consumption*”). Experts noted that consumers’ perceptions regarding euthanasia and transport directly impact the industry’s “*social license to operate*”, demonstrating the importance of timely decisions “*for preserving the social license to have animal agriculture*”.

#### 3.2.11. Supply Chain Considerations

The theme of Supply Chain Considerations was identified across many different areas, primarily as an area that employees needed to understand and as a consideration for decision-making. Knowledge of the animal’s journey after it leaves the dairy was deemed important for employees to understand the impact of shipping compromised cattle; for example, one respondent stated that “*time of transportation, ambient conditions… travel distance*” and understanding that “*loading and unloading processes can worsen… the conditions of a sick animal*” are all important topics for employees to understand. Additionally, experts mentioned “*transportation stress*” and an “*animal’s survival ability during transport*” as topics employees should understand.

Some responses discussed the importance of responsibility throughout the supply chain, with experts sharing misconceptions such as that an animal is “*out of sight out of mind*” and the belief that “*once loaded she isn’t my problem*”. Similarly, experts shared that haulers “*are unaware that they have the final say in where or not to haul a compromised animal*” and subsequently “*they cannot do anything because they do not own the animal*” and “*she walks on the trailer, [so] she will be fine*”. One expert shared that owners may not know that “c*attle that get picked up and leave the dairy can absolutely end up at a slaughterhouse that same day. They also could change hands MULTIPLE times before ending up across the country*”.

#### 3.2.12. Communication

This theme included any mention of communication, as it related to feedback between employees and management and any challenges with communication that may impact employees’ ability to effectively make euthanasia and fitness-for-transport decisions. This theme was found together with the Training and Resources theme in many of the questions related to needs. Experts mentioned language barriers impacting Communication, including that “*many [employees] do not read, cannot read, and may or may not be able to write more than their name*”. Respondents emphasized the importance of having training and resources translated to appropriate languages that are comprehensible for employees such as “*English, Spanish, and Kiiche. (sic)*”. One respondent identified that there is a “*complexity of medical terms*” associated with these end-of-life decisions which can become even more difficult to translate when multiple languages are commonly spoken. Beyond the language component, end-of-life decisions can be a “*difficult topic to talk about*” which may contribute to challenges with Communication. Communication with the supply chain was also discussed. Several responses were shared that related to whether or not the supply chain sectors communicate with one another about the expectations of cows’ conditions (i.e., “*do slaughterhouses communicate expectations of animal welfare with suppliers?*”).

## 4. Discussion

End-of-life decision-making is a critical component of ensuring that animal welfare is maintained throughout a dairy cow’s life. In the dairy industry, end-of-life decisions begin on the dairy, but the responsibility for ensuring the welfare of dairy cattle throughout this time is shared by the entire supply chain including auction markets, transporters, cattle buyers, and slaughter companies [4]. Additionally, animal welfare is complex and multifaceted, including characteristics that often result in different perspectives about what good welfare means between stakeholder groups [33,34,35,36]. Improving end-of-life decisions, which include culling, fitness-for-transport, and euthanasia decisions, is challenging because there is often not a straightforward solution of what is optimal for all stakeholder groups, but further, poor decision-making can have grave consequences for dairy cattle welfare, for the individuals caring for the animals, and for the economic viability and sustainability of the dairy industry. End-of-life decisions are challenging to make and are influenced by many, often competing, factors. This study’s purpose was to utilize expert consultation to identify challenges, factors, and resources that influence end-of-life decision-making for dairy cattle.

The stakeholders recruited for this project had extensive experience within one or more dairy supply chain sectors (e.g., dairy, calf ranch, slaughterhouse, etc.). The respondents all served supportive roles to those who make decisions about and participate in euthanasia and fitness-for-transport activities. Thus, most perspectives reflected in these results are not those of the individuals working directly with the animals at this critical end-of-life juncture but instead are perspectives of the people that help create, distribute, manage, and provide advice and resources to those individuals who are making the often-challenging end-of-life decisions. The largest stakeholder group represented in this study was academia, and most of those individuals were veterinarians, many of whom served in extension roles at a university. Outside academia, there were an additional five practicing veterinarians who participated in the survey. Although there is individual variation across production facilities, veterinarians participate in making recommendations for euthanasia, providing training, and assisting with protocol development on dairies [18,37,38]. In addition to veterinarians, a significant portion of the study population represented industry-affiliated membership organizations or associations that provide support to specific supply chain sectors. There were also some representatives from specific companies that provide technical support in the area of animal care and handling. As individuals working directly with stakeholders in a supportive capacity, the study respondents brought broad perspectives of challenges they encounter while working with different parts of the supply chain around these end-of-life decisions.

It is important to note that in the United States the majority of agricultural workers are foreign-born, facing unique challenges such as language barriers, cultural differences, limited access to legal and social services, and experiencing low income [39]. These factors can significantly influence their decision-making processes and their overall well-being. Recognizing these challenges is crucial for developing effective policies and programs that support agricultural workers and promote sustainable agriculture, including those related to end-of-life decision-making for dairy cattle.

Several questions were asked related to knowledge, training program content, and resources needed to make and execute euthanasia and fitness-for-transport decisions. There was a clear interconnectedness of the themes in the responses and a large breadth of themes within many of them, reinforcing the complexity of these end-of-life decisions. The survey included distinct questions about euthanasia and fitness-for-transport training separately, but many of the responses for both types of training were similar. Cattle health considerations play a clear role in making informed end-of-life decisions, as reflected in responses to questions about subjects that caretakers should understand and the content of training for euthanasia and fitness-for-transport decisions. The two most common themes, generally found together, in all of these questions were Cattle Health Management and Decision-Making; respondents provided detailed information about what cattle health knowledge caretakers should have in order to make informed end-of-life decisions (e.g., “*body condition scoring*”, “*withdrawal times observed for meat and milk*”, “*mobility scoring*”, “*signs of suffering*”, and “*common animal health conditions*”). This clear need for an understanding of cattle health emphasizes the importance of a relationship with a veterinarian. Although veterinarians are cattle health experts relied upon for herd health support, past research indicates that veterinarian involvement in euthanasia decision-making, performance, and training is variable across dairies [18,19,23,40]. There is limited information about the role veterinarians play in training across different supply chain sectors. Similarly, there is very little published information about who is involved in fitness-for-transport decisions for dairy cattle [41], but it has been suggested that veterinarians should have a role in making fitness-for-transport decisions [42]. The knowledge base that respondents indicated is needed to make timely end-of-life decisions, although logical, was vast. On-farm efforts should evaluate euthanasia and fitness-for-transport training to ensure the content included is comprehensive. Additionally, the critical need for decision-making tools (i.e., decision trees) was mentioned across questions. Although there are guidelines for making decisions, there are few widely recognized resources that focus on providing decision tree frameworks to individuals making these difficult decisions across the supply chain.

Training is a critical component to developing animal caretakers’ skills and knowledge [43], and generally livestock care program standards and audits require training for individuals working with animals (e.g., [12,13,14]). Interestingly, despite the general acceptance that training is important for animal caretakers, NAHMS reported that only 20% of participating dairy operations provided euthanasia training to employees [8]. Similarly, only half (50.7%) of the dairies participating in a Canadian cross-sectional study indicated having at least one person on the farm trained in euthanasia [23]. In other studies, across livestock species, animal caretakers [20,23,44] and veterinarians [45] have indicated a desire to participate in or deliver more training in euthanasia specifically, thus identifying an opportunity to enhance training opportunities. Similar information about training related to the fitness for transport of dairy cattle is not available, but the need for fitness-for-transport training for farmers, veterinarians, and transporters has been identified [42,46].

Particularly as it related to euthanasia training content, there were many mentions of knowing “how to” perform the procedure, having the appropriate tools, and knowing how to use them. Many of these comments were contextualized around human safety in operating the equipment. Interestingly, there was little to no mention of the need for training content related to mental wellness while performing euthanasia. In both a study with swine caretakers [44] and swine veterinarians [45], less than half of the participants in each study indicated that euthanasia training included components related to strategies for dealing with “emotional wellness” and “personal stress”. The recognition of mental wellness as a needed area of attention for livestock caretakers who have to perform difficult procedures is being more frequently identified as an area of need [20,44,45,47] and thus it is surprising that its mention did not appear in the responses to the current survey more frequently.

There were many different media mentioned in the responses related to the best methods of communicating needed content (i.e., training material) and needed resources to be able to make end-of-life decisions. Different resources such as flow-charts, posters, visual aids, and decision trees including contacts for support were all identified as helpful tools and resources to use for sharing content with caretakers. There have been some studies in the livestock sector exploring the use of multi-media approaches to train caretakers about important procedures, such as euthanasia, that have been successful at increasing knowledge [48,49,50,51]. Additionally, specific training programs were identified in the responses, such as the FARM program [12], Calf Care Quality Assurance [52], and Beef Quality Assurance [24], illustrating that these training programs are used and supported by those working with the dairy supply chain. There is an opportunity to further develop educational resources and build upon the foundational programs that exist.

Associated with these mentions of specific training programs and types of training resources was a clear identification of the need for effective communication. Effective communication in the survey responses was often explained as “*in-person*”, with many mentions of training needing to be delivered in “*the caregivers’ primary language*”. Livestock caretakers should be, and often prefer to be [53], trained in their native language to ensure that protocols and procedures are understood [48,54], and although this seems like a straightforward and critical component of an effective training program, this is not always the case [55]. Although language is a significant barrier to effective communication with a diverse work force (reviewed by [56]), it is critical to also address other social and cultural differences outside language [48]. A lack of attention to cultural differences (i.e., values, beliefs, history, practices, etc.) in training programs can cause challenges with comprehension and therefore can have significant consequences for worker safety; this has been studied more extensively in the construction industry with a focus on Hispanic workers [57]. Thus, it is critical to have a culturally responsive approach to animal care training for livestock caretakers [58].

Respondents were asked to identify challenges associated with decision-making and the ability to make decisions for euthanasia and fitness for transport. Training and Resources was the most mentioned theme in the responses to these questions. Respondents consistently mentioned that the lack of training (e.g., “*lack of training*” and “*trained labor*”) and/or proper equipment (e.g., “*not having the right equipment*” and “*knowledge of how to use the equipment*”) for performing these job tasks were significant obstacles. As previously discussed, training is critical both to ensure proper animal care but also to make sure employees understand expectations, are satisfied with their jobs, and stay safe. Despite training being a required component of many on-farm and in-plant assessments and audits [12,13,14], there is still an opportunity to enhance training opportunities offered to employees. As mentioned, several studies have explored the benefits of multi-media training approaches related to animal care practices [48,49,50,51]. Additionally, there have been some efforts to craft training opportunities for dairy caretakers that utilize experiential learning techniques to enhance critical-thinking and problem-solving [59]. Again, there has been little work specifically targeted at auction markets and slaughter plants. There is an opportunity in the dairy industry for the development of more and diverse training and continuing education opportunities for caretakers.

Statements related to Company Culture and Communication were also prevalent across responses to questions related to barriers and challenges to decision-making and the performance of end-of-life outcomes. Across all these themes, respondents consistently stated that unclear expectations, a lack of authority to make decisions, and a lack of communication often impacted a caretaker’s ability to perform job tasks in these critical areas. Many respondents shared that a challenge with communication about euthanasia and fitness-for-transport procedures and decisions was Company Culture. Respondents specifically mentioned the lack of delegation and authorization to make decisions as challenges for employees. Respondents also mentioned that employees may be concerned with being blamed about making decisions that management does not agree with and fear the risk of losing their job. Job insecurity can lead to decreased employee psychological well-being, physical health [60], and even job performance [61]. Both the lack of support and pressure from upper management were listed as barriers to timely decision-making as well. While these responses are perceptions based on the respondents’ experience, the prevalence of this sentiment highlights a potential communication breakdown. Even if not universally true, the widespread feeling of a lack of both support and clear expectations indicates a need to address communication around euthanasia and transport decisions. Additionally, past studies have shown a positive relationship between perceived organizational support and job satisfaction [62,63,64] again emphasizing the importance of company culture to employee retention.

A question about misconceptions that employees may have related to end-of-life decisions was included in the survey, and although several themes were present in responses, the dominant theme was Supply Chain Considerations. There was an overwhelming sentiment that caretakers across supply chain sectors often do not fully comprehend the journey that cull dairy cattle take to their final destination once they leave their place of origin. Edwards-Callaway et al. [4] identified that this lack of understanding of the entire journey of a cull cow could contribute to some of the poor decision-making related to fitness for transport. Marshall et al. [41] conducted a study with dairy farmers in Ontario and found that, for a portion of their cull cows, some farmers did not know their own cows’ destination for transport. There is little published information documenting the entire journey that a cull cow takes once it leaves the dairy in the United States. The NAHMS program provides some information about distances culled dairy cows travel once they leave the farm to their initial destination [8] and the NCBA National Beef Quality Audit (NBQA) provides information about the distance culled dairy cattle travel from their last place of origin to the plant [15], but neither one provides a comprehensive overview of the entire travel time (e.g., both in the truck and at other locations along the way such as auction markets) or distance. Although some dairy cattle are shipped directly to a slaughter plant [8], the majority are sold first through an auction market and may be in the marketing system for several days prior to being slaughtered (e.g., 82 ± 46 h; [17]). Additionally, there is little shared information across supply chain sectors (i.e., dairy to auction market to slaughter plant) about the condition of culled dairy cattle as they move through the supply chain, likely exacerbating the lack of awareness of how the marketing process can impact cows’ conditions. Research has demonstrated that shipping from the dairy to slaughter increased the odds of poor fitness for transport [17]. In the current survey, respondents also noted that many individuals working with cattle likely do not know that ownership changes as the dairy cattle move to their final destination. The change in ownership can create challenges for holding people accountable for animals’ conditions. Some respondents indicated that a lack of communication regarding the expectations of the supply chain (e.g., “*it[’]s hard to communicate with auction customers*” and “*do slaughterhouses communicate expectations of animal welfare with suppliers?*”) was an obstacle. Finding opportunities to incorporate information about the entire marketing process and supply chain connections may help better inform caretakers across sectors about the importance of end-of-life decision-making for dairy cattle welfare.

One significant factor influencing the end-of-life outcomes for dairy cattle that was mentioned across several survey questions, both described as an incentive and a disincentive, was the economic impact of end-of-life decisions. Edwards-Callaway et al. [4] describes the financial incentives that are associated with shipping compromised cows to slaughter and which underline some poor decisions. In short, there is no significant disincentive, outside concern for a cow’s welfare, for shipping, selling, or purchasing this class of animals, but there is often actually a financial incentive (e.g., the revenue from selling the animal, not having to pay for euthanasia or disposal fees) to do so. Respondents in this survey suggested using economic incentives as a motivator for individuals to make better decisions for cow welfare. Some responses suggested financially rewarding employees for good decisions (e.g., “*bonuses tied to having no down animals*”) or not penalizing these decisions (e.g., “*no deductions for euthanasia or non transported animals to the employees*”) and others indicated there should be negative consequences for poor decisions (e.g., “*penalty if an animal shouldn’t have been shipped or should have been euthanized*”).

The relationship between employee performance and incentives has been explored across various types of organizations (including in meta-analyses by [65,66,67]). Incentivizing making good welfare decisions is likely occurring at some level across the dairy supply chain and its implementation is probably variable across facilities; there is little publicly available information about both how this is performed in practice and the documented success of these types of incentive programs. Condly et al. [66] conducted a meta-analysis of research studies exploring the effects of incentives on workplace performance and reported that money resulted in greater performance gains than non-monetary rewards (e.g., gift cards). Condly et al. [66] also reported that team-based incentives had a greater effect on performance; in a separate meta-analysis, Garbers and Konradt [67] also reported a positive effect of team-based rewards on performance. Considering that there are often several individuals involved in making end-of-life decisions, team-based incentive frameworks for making end-of-life decisions could be a beneficial option to improve performance. Condly et al. [66] reported that incentive-based performance gain is greater in physical tasks than mental tasks, which could be related to both the difficulty in measuring performance associated with cognitive tasks and the ease (or difficulty) in increasing performance associated with the different types of work. Considering that end-of-life decision-making has a clear mental component, work needs to be performed to understand how incentivizing these decisions may best be implemented. Including animal welfare outcomes as key performance indicators linked to employee job performance is an area that deserves further exploration.

Respondents were asked to identify personal beliefs that may impact end-of-life decision-making, and a large range of different impacting factors were included in responses spanning from employee emotions towards animals to feelings about their job responsibilities to religious beliefs. One of the most prominent concepts that was shared was empathy for animals, i.e., the ability to understand and recognize the feelings of other beings. How a person cares (or does not care) for animals can impact euthanasia and fitness-for-transport decisions. Past studies with both dairy and swine caretakers have demonstrated that caretakers’ empathy and compassion towards animals impacts how they make end-of-life decisions for the animals under their care [20,68,69]. Caretakers across the dairy supply chain have been tasked with caring for animals and keeping them alive and thus when asked to end their lives, even if it is the best option for the animal, there is often moral stress associated with these decisions. This was captured in the current study in comments such as “*[my] job is to save them not kill them*”, “*if there is still a chance, she is going to recover we have to keep trying*” and “*I need to give them a chance to get better*”. It should be reiterated that these responses are from individuals working within the dairy industry representing their thoughts about challenges that caretakers may face, not comments from caretakers themselves. Walker et al. [9] suggests that the bond between dairy caretakers and their cows can be a barrier to timely euthanasia decisions. It is necessary to recognize, understand, and address the feelings of caretakers across the dairy supply chain in order to help support them in making these often-difficult decisions.

Company Culture was again mentioned from an organizational perspective as a potential barrier to decision-making. Respondents highlighted how organizational leadership can impact these end-of-life decisions in a variety of ways. Comments were mostly related to a lack of providing sufficient structure and resources for employees to make informed decisions, i.e., “*insufficient oversight*”, “*inadequate management support*”, and a “*lack of clear rules about who is responsible for a decision and for action*”. Establishing a culture of care in a livestock operation is essential to success. It is hypothesized that some companies are very intentional about communicating the importance of animal welfare; others, although supportive, may not adequately elevate this importance, while others may not prioritize welfare procedures and policies. Although not mentioned directly in responses to the survey questions, it is also important to consider industry culture around animal welfare. Particularly in the case of culled dairy cattle, there are many stakeholders that impact the end-of-life outcome for these animals (i.e., dairy operators, transporters, auction market owners, cattle buyers, etc.). As of yet, intentional collaborative initiatives have not been formed to address end-of-life decision-making in dairy cattle in the United States. Fernandes et al. [70] emphasized the importance of involving stakeholders in discussions about animal welfare as these collaborative networks generate shared priorities and governance in addition to promoting productive communication. Collaborative networks that cut across supply chain sectors bring together a diversity of expertise, knowledge, and resources that are valuable in promoting innovative and creative thinking about complex issues [71] such as end-of-life decision-making for dairy cattle. Additionally, by working together on a common challenge, alignment to a united vision can be established which ultimately will lead to shared ownership of the problem and the solution. Recognizing the impact that organizational leadership, both at a company and industry level, has on how employees make decisions is critical to improving end-of-life decision-making for dairy cattle.

Another important factor that was mentioned as an influential factor was Consumer Perceptions. Poor end-of-life decisions not only compromise animal welfare but also put the dairy industry at risk by compromising public trust in livestock production. Animal welfare receives significant public attention and increasingly influences societal perception of agricultural practices and food production [72,73,74,75]. For example, dairy cattle that require euthanasia but that are not euthanized pose a risk for consumer confidence in the dairy industry [9]. In a willingness-to-pay study, consumers ranked prompt treatment and/or euthanasia as being a component of dairy management that would be effective at indicating good animal welfare [76] indicating the importance and visibility this area has for consumers. In recent years, animal activists have used undercover videos to further anti-agriculture agendas, and some undercover videos depict compromised cows and calves that should have been euthanized (e.g., [77]). Compromised dairy cows and calves at slaughter plants have also been the focus of undercover videos in the past, with one in particular resulting in the largest ground beef recall in the United States [78,79]. These images of animals suffering are a great risk to the sustainability of the dairy industry, emphasizing the priority that needs to be placed on improving end-of-life decisions across the dairy supply chain. In the current survey, respondents noted the importance of timely end-of-life decisions “*for preserving the social license to have animal agriculture*”.

## 5. Conclusions

To our knowledge, this was the first study to gather expert opinions on the variety of factors influencing euthanasia and fitness-for-transport decisions for dairy cattle. Experts emphasized the importance of robust training programs that should include criteria for euthanizing cows or determining cows’ fitness for transport, prognosis for common conditions, responsible personnel, appropriate euthanasia methods, cattle handling, and supply chain considerations (e.g., the distance and duration of transport after leaving the dairy and the number of stops and loading or unloading events). However, factors in addition to training were recognized for their potential to impact euthanasia and fitness-for-transport decisions, including economics, company culture (e.g., empowering caretakers to make timely decisions that prioritize animal welfare), communication, and personal beliefs. Furthermore, experts discussed the importance of all supply chain stakeholders taking responsibility and making timely decisions to ensure good animal welfare. A holistic approach to improving timely euthanasia and fitness-for-transport decisions is therefore necessary.

## Figures and Tables

**Table 1:** Questions from the survey tool. The operation types listed in the brackets for each question were as follows: dairy farms, calf ranches, haulers, auctions, and slaughterhouses. Those options have been deleted from this table for brevity.

1	Please list the topics or subjects that employees of [operation type] should understand to make timely euthanasia decisions.
2	Please list the topics or subjects that employees of [operation type] should understand to make fitness for transport decisions.
3	Please list the most effective channels or methods for communicating with people who make decisions about timely euthanasia and fitness for transport.
4	List the content of topics that a training program on timely euthanasia and fitness for transport for employees of [operation type] should include.
5	List the misconceptions the employees of [operation type] may have in making decisions about timely euthanasia and fitness for transport.
6	List the challenges or barriers that employees of [operation type] encounter that might influence their decision-making of timely euthanasia and fitness to transport.
7	List the challenges or barriers that employees of [operation type] encounter that might influence their communication of timely euthanasia and fitness to transport.
8	List the challenges or barriers that employees of [operation type] encounter that might influence their ability to perform timely euthanasia and determine fitness to transport.
9	List the resources that [operation type] should have for making timely euthanasia and fitness for transport decisions.
10	List the potential incentives/motivators that may improve the implementation of practices of timely euthanasia and appropriate fitness for transport on [operation type].
11	List the potential personal beliefs that may impact the implementation of practices for timely euthanasia and fitness for transport on [operation type].
12	List the potential social factors that may impact the implementation of practices for timely euthanasia and fitness for transport on [operation type].
13	List the potential organizational factors that may impact the implementation of practices for timely euthanasia and fitness for transport on [operation type].
14	List the potential economic factors that may impact the implementation of practices for timely euthanasia and fitness for transport on [operation type].
15	List the potential regulatory factors that may impact the implementation of practices for timely euthanasia and fitness for transport on [operation type].
16	List other factors regarding the dairy supply chain that you think may be relevant to timely euthanasia and fitness for transport on [operation type].
17	Please use this space below to input any additional comments or suggestions.

**Table 2:** Theme, definition, and example excerpts from responses utilized in thematic analysis.

Theme	Definition	Example Excerpts
Training and Resources	Comments relating to the methods of training, having or not having skills/knowledge to perform duties, physical equipment and tools used in euthanasia, protocols and SOPs, records or written documentation of individual animals, the availability or lack thereof of methods for disposing of dead animals, and time or labor availability to complete one’s job.	“*Hands-on and case-based learning often is the most impactful*”. “*…availability of properly maintained euthanasia tools*”. “*…treatment protocols, euthanasia protocols…*” “*…availability of person responsible for the decision making*”.
Cattle Health Management	Statements regarding animal health, illness, prognosis, identification, diseases, treatment, veterinary evaluation or the administration of treatment, and the care of animals.	“*Recognizing normal versus abnormal health conditions*”. “*…disease prognosis…*” “*…likely real-time necessitating an understanding of suffering/well-being and realistic treatment opportunities*”. “*…the importance of timely vet evaluation*”.
Decision-Making Criteria	References to criteria that influence the decision-making process including animal-based scoring, withdrawal periods, unacceptable animal conditions, organization decision-making trees, reasons or the why behind one’s decision, and the chain-of-command for making decisions.	“*…who to consult if there are questions about euthanizing cattle, primary/secondary decision-maker and person responsible for euthanizing cattle*”. “*…dairy body condition score, mobility score…*” “*…a decision tree of decision makers for these topics*”. “*…reasons for euthanasia, reasons for condemnation of carcasses at slaughter…*”
Company Culture	Anything relating to the organization’s structure, roles and expectations of employees, authority, confidence, trust, organization priorities, reputation, and overall culture. This is restricted to the organization itself and does not expand to the supply chain or beyond the direct organization.	“*…authority from their employer to make those decisions…*” “*…lack of trust between workers and managers…*” “*Fear of being blamed for the animal’s condition…*” “*…understand that a cull cow going to slaughter still has YOUR tag in it’s ear that is advertising YOUR husbandry practices…*”
Personal Beliefs	Mentions of employees’ culture, emotions towards animals, feelings of job duties, religious beliefs, the sacredness of an animal, and feelings of failure due to taking a life (e.g., a waste of life).	“*…evil to take a life…*” “*Concern about perception of failure…*” “*…compassion-fatigue…*” “*…not being asked about their feeling of performing euthanasia…*”
Human Well-Being	Statements referring to the employee’s safety, the use of firearm safety, consumer and public health safety, food supply safety, and the well-being of the employee or consumer.	“*Lack of workplace culture where animal or worker welfare is a priority*”. “*…how to be safe, firearm safety…*” “*Food Safety—how to ID animals who must be removed from the food chain in a timely manner*”. “*…employee safety and proper handling of euthanasia equipment…*”
Animal Welfare	Anything regarding the animal’s quality/value of life, stress, pain, suffering, well-being, happiness, overall demeanor, abnormal behavior, or the lack thereof.	“*Cattle don’t feel pain/stress*”. “*pain that can not be managed; animal’s welfare/wellbeing*” “*Animal distress (i.e., extreme weather conditions, udder condition)*” “*If they wait too long to make the decision to euthanize an animal, it could be too late to transport it, and it won’t be able to cope with the stress*”
Economics	Mentions of money, financial gain/loss, considerations of cost efficiency, loss of business, selling/building animals, and profitability.	“*Selling an animal at any price is more profitable than euthanizing it so the organization frowns upon euthanizing*”. “*If animals die on farm due to delayed removal (while still marketable), there is an obvious economic loss. If animals become non-ambulatory during transport there is an obvious economic loss as well (and potential cost)*”. “*Financial loss and cost of euthanizing calves…*” “*not to suffer complete economic loss of having to euthanize a cow on farm., Pressure to get paid for transporting a cow, Pressure not to lose a sale*”
Guidelines and Inspections	References to or the lack thereof of standards, industry organization guidelines, audits, federal inspections, laws, and third party, industry, or internal inspections.	“*…euthanasia standards (example: AABP guidelines)…*” “*No on farm regs*”. “*More welfare audits of auctions that include evaluation of number of down, injured animals and euthanasia*”. “*More restrictions on movements and fines for not following the rules*”.
Consumer Perceptions	Mentions of consumers’ perceptions, thoughts, and ideas about animal agriculture or the dairy supply chain. This includes social media, random photos, and animal activist group videos.	“*Bad publicity and boycotts or bans*”. “*Consumer perception-is it positive or negative in euthanizing on farm when warranted*”. “*Public would react negatively if animal allowed to suffer. Timely euthanasia is important for preserving the social license to have animal agriculture*”. “*Inappropriate euthanasia decisions might affect the social perception of the dairy industry affecting consumption*”.
Supply Chain Considerations	Statements regarding animal ownership, retained animal ownership, variety of stakeholders, accountability and responsibility for animal throughout supply chain, accountability back to the original owner, the needs of animals during hauling, the perceptions and thoughts of supply chain members, and pressure from supply chain members on one another. This all relates to after the animal leaves the facility.	“*Retained ownership along the value chain is one of the few ways I believe that owners are motivated to truly do the right thing*”. “*Develop the list of no-go’s, communicate to industry, and then hold entire chain accountable*”. “*…supply chain (where is the animal going, how long will it take, what is it like to ride in a trailer)…*” “*…lack of understanding of the supply chain…*”
Communication	Anything regarding feedback or lack thereof, consultation, communication or lack thereof between the employee and employer/organization, language, language differences, and primary language. This can include communication with a veterinarian as well.	“*It would be great to have more transparency about how long it takes for cattle to move from point A to point B. Especially new baby calves*”. “*communicating with employees if an animal was denied at the auction barn or slaughterhouse- feedback!*” “*What is the communication like amongst employees, their peers, from them to their supervisor, and from the leadership to owners?*”

## Data Availability

Data are available upon request to the corresponding author due to privacy restrictions.
